# Biphasic Modulation of Absence Seizures by Lisuride in GAERS: Dissecting the Role of 5-HT_2A_ Receptors

**DOI:** 10.3390/ijms27219815

**Published:** 2026-11-01

**Authors:** Marcello Venzi, Vincenzo Crunelli, Giuseppe Di Giovanni

**Affiliations:** 1 Neuroscience Division, School of Bioscience, Cardiff University, Museum Avenue, Cardiff CF10 3AX, UK; marcello.venzi@gmail.com; 2 Institute of Pharmacology and Neurosciences, Faculty of Medicine, University of Lisbon, 1649-028 Lisbon, Portugal; 3 College of Medicine, Korea University, Seoul 02841, Republic of Korea; 4 Department of Medical and Surgical Sciences, University of Magna Graecia, 88100 Catanzaro, Italy

**Keywords:** absence epilepsy, GAERS, thalamocortical network, ventrobasal thalamus, spike–wave discharges, in vivo electrophysiology

## Abstract

Serotonergic signalling modulates absence seizures, and activation of 5-HT_2A_ receptors with TCB-2 suppresses spike-and-wave discharges in Genetic Absence Epilepsy Rats from Strasbourg (GAERS). We investigated the effects of lisuride, a serotonergic and dopaminergic ligand with low 5-HT_2A_ receptor signalling efficacy, and examined the contribution of these receptors. Spontaneous seizures were recorded in freely moving male GAERS following systemic administration of lisuride (0.1 or 0.5 mg/kg), with or without pretreatment with the 5-HT_2A_ receptor antagonist MDL11,939. Low-dose lisuride produced a modest pro-absence effect, whereas the higher dose produced a biphasic response: early seizure suppression followed by increased seizure activity. MDL11,939 did not abolish the early suppression, but the late increase observed with lisuride alone was not observed following pretreatment. These findings suggest that the early and late responses involve different mechanisms, with 5-HT_2A_ receptors potentially contributing to the later phase. Lisuride’s effects differ from those reported for the potent 5-HT_2A_ receptor TCB-2, consistent with its broader receptor profile. Further work is needed to determine the contribution of its other targets, including dopaminergic receptors.

## 1. Introduction

Typical absence seizures (ASs) are generalized non-convulsive seizures characterized by transient impairment of consciousness and highly synchronized spike-and-wave discharges (SWDs). Their generation depends on abnormal interactions within cortico-thalamo-cortical networks involving the cerebral cortex, thalamocortical (TC) neurons and the thalamic reticular nucleus [1,2]. Genetic Absence Epilepsy Rats from Strasbourg (GAERS) reproduce the principal electrographic, behavioral and pharmacological features of human absence epilepsy and provide a well-established model for investigating the mechanisms controlling ASs [3].

Serotonin (5-HT) is an important modulator of thalamocortical excitability [4], and recent evidence indicates that several 5-HT receptor subtypes influence AS expression. Activation of 5-HT_2C_ receptors (5-HT_2C_Rs) with the agonist Ro 60-0175 suppresses spontaneous ASs in GAERS and reduces the enhanced tonic GABA_A_ inhibition of ventrobasal (VB) TC neurons [5], further supporting the potential cellular link between serotonergic modulation of thalamic inhibition and seizure control [6,7]. This is consistent with the anti-absence effect of 5-HT_2A_R, as systemic administration of the potent 5-HT_2A_R agonist (4-bromo-3,6-dimethoxybenzocyclobuten-1-yl)methylamine (TCB-2) markedly reduces ASs in GAERS, whereas the selective antagonist MDL11,939 transiently increases seizure activity [8]. Moreover, thalamic 5-HT_2A_R activation by TCB-2 reduces tonic GABA_A_ inhibition in TC neurons and suppresses ASs [9,10].

Nevertheless, 5-HT_2A_Rs can exert opposite effects on thalamic GABAergic signaling in normal conditions, enhancing tonic GABA_A_ inhibition and promoting SWDs in non-epileptic Wistar rats, further supporting the context-dependent regulation of thalamocortical excitability by 5-HT_2A_Rs [11].

The consequences of 5-HT_2A_R activation, however, may also depend on receptor occupancy, intrinsic efficacy, and downstream signaling, and therefore differ considerably among agonists [12,13,14]. This is particularly relevant because classical serotonergic psychedelics exert their characteristic behavioral effects through 5-HT_2A_Rs, yet not all 5-HT_2A_R agonists are psychedelic, with lisuride providing a particular example in this context [15,16].

Importantly, lisuride is not a selective 5-HT_2A_R ligand but has a broad pharmacological profile involving serotonergic and dopaminergic receptors [17].

Lisuride was therefore selected for determining whether the anti-absence effects previously observed with TCB-2, a 5-HT_2A_R agonist with potential hallucinogenic properties [18], could be reproduced by a pharmacologically distinct, non-psychedelic 5-HT_2A_R ligand. This distinction is particularly relevant to the clinical translation of these findings, as potential psychedelic effects pose a significant obstacle to the development of treatments for absence epilepsy, especially in children [19]. Furthermore, given lisuride’s broad receptor profile, the selective 5-HT_2A_R antagonist MDL11,939 [20] was used to determine whether its effects on absence seizures depend on 5-HT2AR activation.

We therefore investigated the effects of lisuride on spontaneous ASs in freely moving male GAERS. Lisuride produced a dose- and time-dependent biphasic response, predominantly affecting seizure duration, with early seizure suppression followed by a delayed increase in seizure activity. MDL11,939 did not affect the early anti-absence effect but reversed the delayed increase in seizure burden and duration, indicating distinct mechanisms underlying the biphasic response.

## 2. Results

### 2.1. Lisuride Produces Dose- and Time-Dependent Modulation of ASs

**Figure 1:**
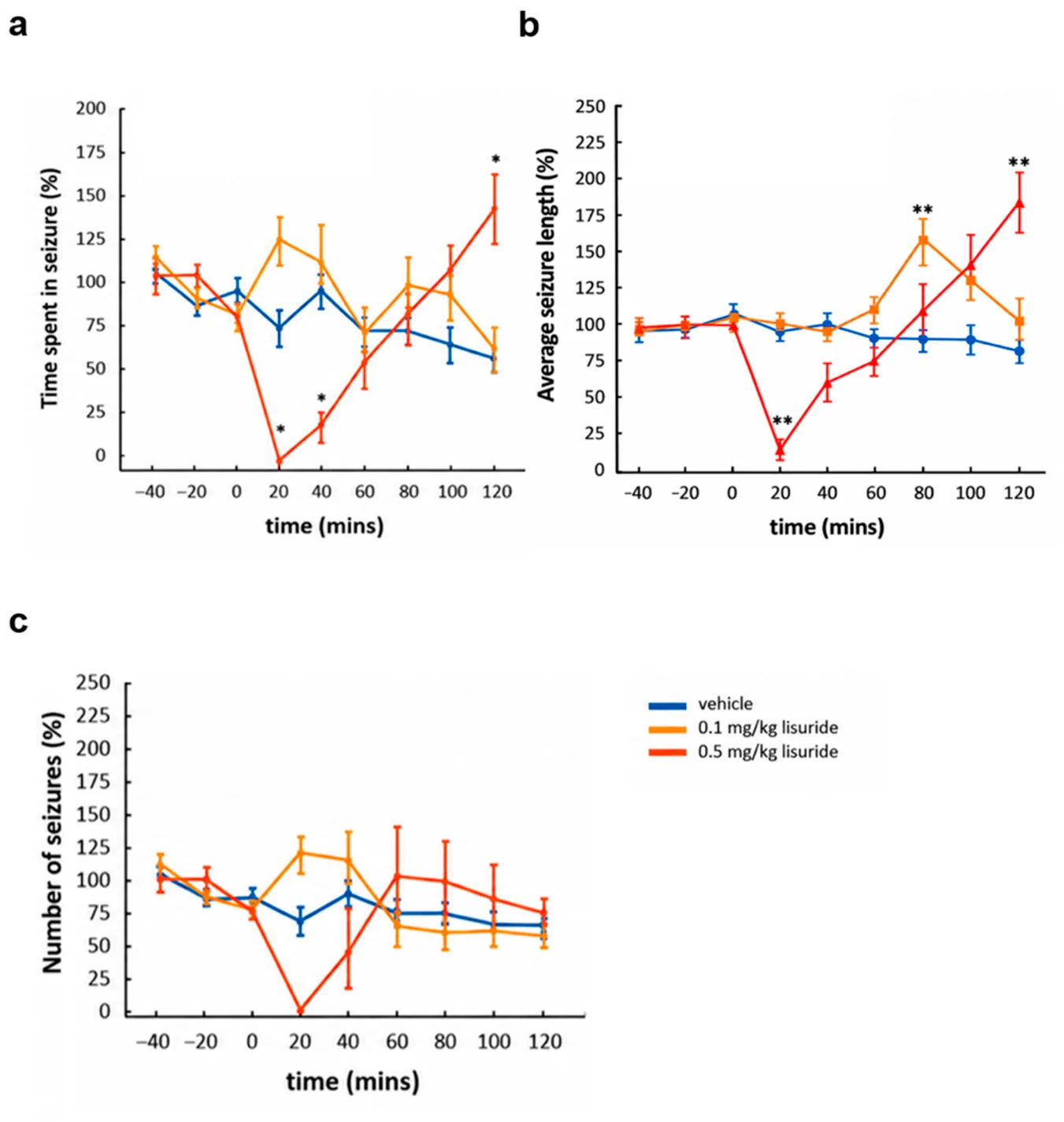
Lisuride produces dose- and time-dependent biphasic modulation of ASs in male GAERS. (**a**–**c**) Time course of spontaneous absence seizure (AS) parameters following systemic administration of vehicle or lisuride (0.1 and 0.5 mg/kg, i.p., *n* = 9 animals per group). (**a**) Time spent in seizures; (**b**) mean seizure duration; (**c**) number of seizures. At 0.1 mg/kg, lisuride produced modest effects, including a significant increase in seizure duration at 80 min. In contrast, 0.5 mg/kg lisuride produced a pronounced biphasic response, characterized by an early suppression of ASs followed by delayed facilitation, predominantly associated with an increase in seizure duration. All values were normalized to the mean of the three 20-min intervals during the pre-treatment control period (−40 to 0 min), defined as 100% for each animal. Lines and bars represent the mean and SEM, respectively. Asterisks indicate significant differences at the indicated time points: * *p* < 0.05 and ** *p* < 0.01. Full statistical results from the two-way ANOVA, including the main effects of treatment and time, their interaction, and Dunnett’s post hoc comparisons are provided in Supplementary Table S1A.

Systemic administration of lisuride produced dose-, time and time x treatment-dependent effects on spontaneous ASs in male GAERS compared to controls (two-way ANOVA; Supplementary Table S1A, *n* = 9 animals per group). At 0.1 mg/kg i.p., lisuride produced a significant overall increase in time spent in seizure (31.9%, *p* < 0.05) and average seizure length (26.9%, *p* < 0.05) without significantly affecting seizure number. At individual time points, time spent in seizure was unchanged, whereas average seizure length was significantly increased at 80 min (90.7%, *p* < 0.01). In contrast, 0.5 mg/kg i.p. lisuride produced a pronounced biphasic response (Figure 1a–c). Time spent in seizure was reduced by 99.2% at 20 min and 82.2% at 40 min (both *p* < 0.05), followed by a significant 118.4% increase at 120 min (*p* < 0.05). Average seizure length showed a similar profile, decreasing by 85.1% at 20 min (*p* < 0.01) and increasing by 101.9% at 120 min (*p* < 0.01). Seizure number was not significantly altered at individual time points, indicating that the delayed increase in seizure burden was predominantly associated with prolonged ASs.

### 2.2. 5-HT_2A_ Receptor Blockade Differentially Modifies the Early and Late Effects of Lisuride

**Figure 2:**
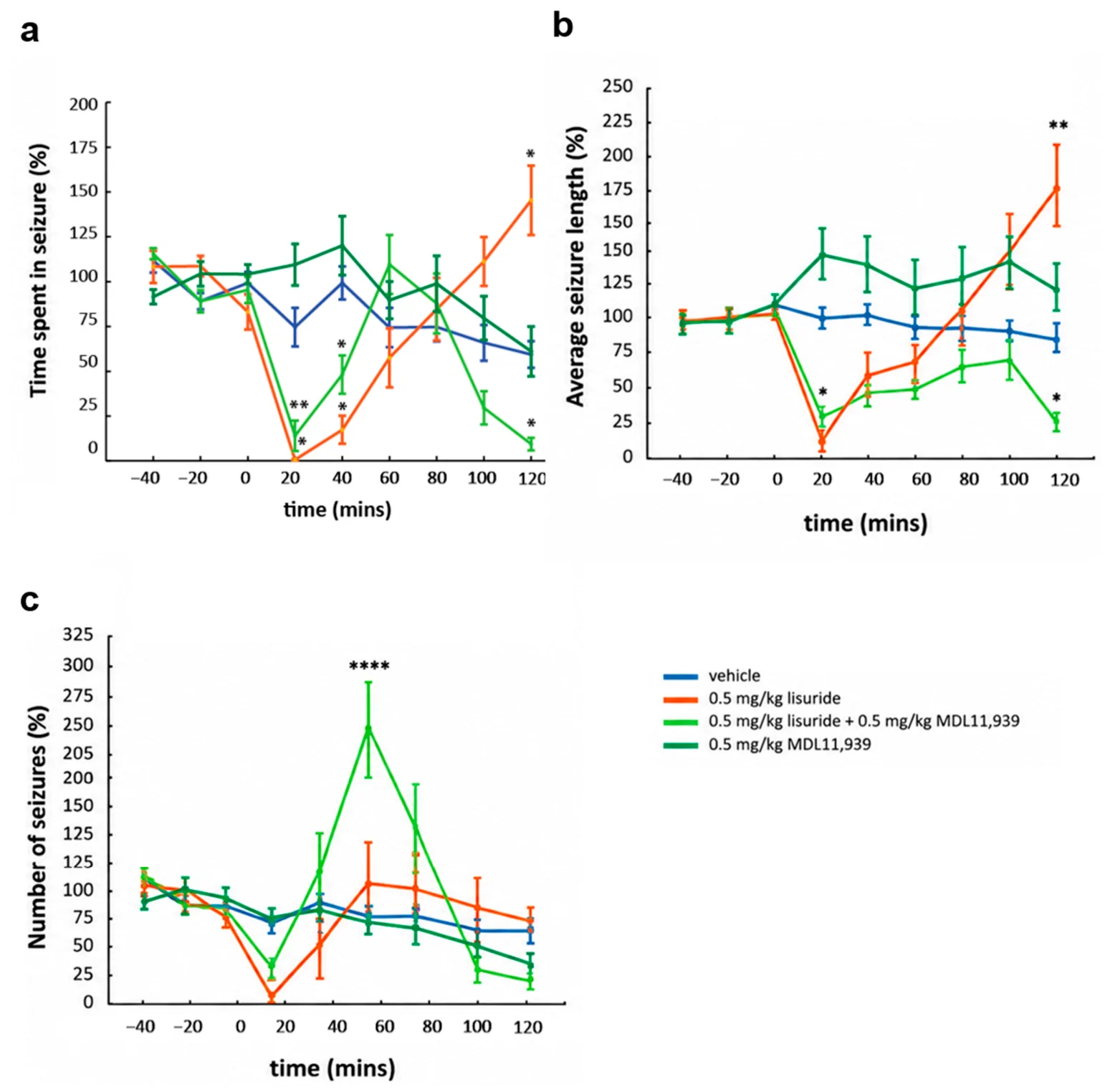
The 5-HT_2A_ receptor antagonist MDL11,939 differentially modulates the biphasic effects of lisuride on ASs in male GAERS. (**a**–**c**) Time course of spontaneous absence seizure (AS) parameters following systemic administration of vehicle, lisuride (0.5 mg/kg, i.p.), MDL11,939 (0.5 mg/kg, i.p.), or lisuride (0.5 mg/kg, i.p.) following 10 min pretreatment with MDL11,939 (0.5 mg/kg, i.p.) (n = 9 per group). (**a**) Time spent in seizures; (**b**) mean seizure duration; (**c**) number of seizures. Pretreatment with MDL11,939 did not prevent the early anti-absence effect of lisuride but markedly modified its subsequent effects, preventing the late increases in time spent in seizures and seizure duration, and producing a transient increase in seizure number at 60 min. All values were normalized to the mean of the three 20-min intervals during the pre-treatment control period (−40 to 0 min), defined as 100% for each animal. Lines and bars represent the mean and SEM, respectively. Asterisks indicate significant differences at the indicated time points: * *p* < 0.05, ** *p* < 0.01, and **** *p* < 0.0001. Full statistical results from the two-way ANOVA, including the main effects of treatment and time, their interaction, and Dunnett’s post hoc comparisons are provided in Supplementary Table S1B.

To examine the contribution of 5-HT_2A_Rs to lisuride’s effects, male GAERS received the selective 5-HT_2A_R antagonist MDL11,939 (0.5 mg/kg, i.p.) 10 min before lisuride (0.5 mg/kg, i.p.) (Figure 2a–c). The lisuride-only and vehicle groups were the same animals included in the dose–response analysis; separate MDL11,939-only and lisuride-plus-MDL11,939 groups were added (*n* = 9 animals per group). Two-way ANOVA revealed significant effects of treatment and time, as well as a treatment × time interaction, on spontaneous absence seizures (Supplementary Table S1B). MDL11,939 did not prevent the early anti-absence effect of lisuride. Following combined treatment, time spent in seizure was reduced by 82.2% at 20 min and 54.4% at 40 min (both *p* < 0.05), while average seizure length was reduced by 74.6% at 20 min (*p* < 0.05). Thus, substantial early seizure suppression persisted during 5-HT_2A_R blockade.

In contrast, MDL11,939 reversed the delayed response to lisuride. At 120 min, lisuride alone increased time spent in seizure by 127.0% (*p* < 0.001) and average seizure length by 95.0% (*p* < 0.01), whereas following MDL11,939 pretreatment, these measures were reduced by 85.4% and 73.0%, respectively (both *p* < 0.05). Combined treatment also produced a pronounced transient increase in seizure number at 60 min (257.2%, *p* < 0.0001), with no significant changes at other time points. MDL11,939 alone did not significantly alter time spent in seizure or seizure number at any individual time point, although a significant overall effect on average seizure length was observed (*p* < 0.01) (Figure 2a–c).

Notably, lisuride was associated with agitation and erratic locomotor activity, which persisted following MDL11,939 pretreatment; however, these behaviors were not systematically quantified.

## 3. Discussion

This study demonstrates that systemic lisuride produces a dose- and time-dependent biphasic modulation of spontaneous ASs in male GAERS. While 0.1 mg/kg produced modest pro-absence effects, 0.5 mg/kg caused profound early suppression of SWDs, followed by facilitation during the second hour. The early effect involved reductions in both seizure number and duration, whereas the delayed increase in seizure burden predominantly reflected prolonged SWDs. MDL11,939 did not prevent the early suppression but prevented the delayed response, suggesting that distinct receptor mechanisms may contribute to the two phases of lisuride action.

These findings contrast with an earlier report in the same model of male GAERS, which reported negligible effects of lisuride at doses of 0.25–0.5 mg/kg but nearly complete suppression of SWDs at 1 mg/kg, a dose associated with mortality [21]. This apparent discrepancy may reflect differences in experimental design, including the lack of habituation and the shorter baseline, and particularly, post-treatment observation periods in the earlier study. Indeed, the facilitatory effect observed here emerged only during the second hour, a period not examined by Warter et al. (1988) [21]. The possibility that differences in recording protocols account for these discrepancies is further supported by the limited involvement of 5-HT_2_Rs in ASs suggested in earlier work by the same group [22], whereas subsequent studies using the same experimental design as the present work demonstrated robust 5-HT_2A_R-mediated modulation of ASs [8,9].

Biphasic effects of lisuride are not restricted to seizures but have also been observed in locomotor activity, with suppression at low doses and stimulation at higher doses, effects attributed to its complex dopaminergic and serotonergic pharmacology [23,24]. Dopaminergic and serotonergic systems are closely interconnected and jointly modulate many physiological and pathological processes including epilepsy [25,26]. In GAERS, nigrostriatal dopaminergic degeneration prolongs SWDs and alters serotonergic transmission [27]. However, the contribution of dopaminergic–serotonergic interaction to the modulation of ASs remains to be directly demonstrated.

The net effect of lisuride may therefore reflect a time-dependent interplay between dopaminergic and serotonergic actions, rather than a single neurotransmitter mechanism.

Dopaminergic mechanisms may contribute to lisuride’s early anti-absence effect, consistent with its high affinity for D2-like receptors and previous evidence that DA receptor agonists suppress SWDs in GAERS [21]. Furthermore, D2R activation reduces the enhanced tonic GABA_A_ inhibition of VB TC neurons, a key pathophysiological mechanism contributing to AS generation in GAERS [7,28]. However, lisuride is also a potent 5-HT_1A_R agonist [15], and activation of these receptors has been shown to facilitate SWDs in WAG/Rij rats [29]. Thus, lisuride’s early anti-absence effect may reflect D2-like receptor mediated mechanisms overriding potential pro-absence effects of 5-HT_1A_R receptor activation, a hypothesis requiring confirmation with selective antagonists.

A major contribution of 5-HT_2A_Rs to lisuride’s early anti-absence effect appears unlikely, as this effect was not prevented by the highly selective 5-HT_2A_R antagonist MDL11,939, which exhibits negligible affinity for 5-HT_1A_Rs [30]. Importantly, the same dose of MDL11,939 effectively antagonized the effects of the potent 5-HT_2A_R agonist TCB-2 in GAERS [8]. Nevertheless, incomplete 5-HT_2A_R blockade cannot be entirely excluded, as only one dose of MDL11,939 was tested in the present study, and neither receptor occupancy nor the duration of functional antagonism was directly assessed.

Notably, selective activation of 5-HT_2A_Rs by TCB-2 produces a marked, dose-dependent suppression of ASs in GAERS under the same experimental conditions used in the present study [8,9]. Unlike lisuride, however, TCB-2 produces a more sustained anti-absence effect without a biphasic response, highlighting the distinct pharmacological profiles of these two compounds [8,9].

Lisuride has been described as a non-hallucinogenic 5-HT_2A_R agonist, a pharmacological profile previously attributed to biased agonism [31]. Its potential anti-absence effects are therefore of particular interest from a translational perspective, as the hallucinogenic properties of classical psychedelics represent a significant obstacle to the clinical development of 5-HT_2A_R agonists for the treatment of absence epilepsy, especially in pediatric populations [19].

More recent evidence indicates that lisuride exhibits relatively low intrinsic efficacy across multiple 5-HT_2A_R signalling pathways, including Gq- and β-arrestin2-mediated signalling. Accordingly, as a low-efficacy partial agonist, lisuride may exhibit functional antagonist-like effects in the presence of higher-efficacy agonists [15,32,33].

Surprisingly, the delayed pro-absence effect of lisuride was prevented by the selective 5-HT_2A_R antagonist MDL11,939. This finding is particularly intriguing given that selective 5-HT_2A_R activation exerts anti-absence, as demonstrated by the suppression of ASs in GAERS following both systemic and intrathalamic administration of TCB-2 [8,9]. The delayed pro-absence response to lisuride may therefore involve a 5-HT_2A_R-sensitive mechanism whose functional consequences depend on the prevailing thalamocortical network state and interactions with other receptor-mediated signaling pathways, potentially influenced by lisuride’s low intrinsic efficacy at 5-HT_2A_Rs [15]. Notably, intra-VB administration of TCB-2 induces ASs in non-epileptic Wistar rats [11], in contrast to its anti-absence effects in GAERS [9]. These opposing effects highlight the network-state-dependent nature of 5-HT_2A_R-mediated modulation of thalamocortical oscillations.

A key finding of the present study is the differential effect of lisuride on seizure number and duration during the two phases of its action. At 20 min, lisuride reduced both parameters, whereas the delayed increase in seizure burden at 120 min predominantly reflected prolonged SWDs without a corresponding increase in seizure number. This suggests that lisuride may differentially modulate mechanisms governing SWD initiation and maintenance/termination, consistent with current concepts of AS generation. SWD initiation involves cortical networks and the recruitment of cortico-thalamo-cortical circuits [2,34]. Recent optogenetic evidence further shows that VB thalamocortical neurons can both initiate and terminate ASs depending on the network state [35], supporting partially distinct mechanisms controlling AS occurrence and maintenance/termination. These processes may be differentially modulated by DA and 5-HT, both of which are potent modulators of thalamic function. Consistent with this interpretation, MDL11,939 did not prevent the early reduction in seizure number and duration but prevented the delayed lisuride-induced prolongation of ASs. Interestingly, combined administration of lisuride and MDL11,939 also produced a transient increase in seizure number at 60 min without a corresponding significant increase in mean seizure duration. This unexpected effect might reflect the unmasking of potential 5-HT_1A_R-mediated pro-absence actions of lisuride following 5-HT_2A_R blockade, thereby transiently facilitating seizure initiation within thalamocortical networks. Thus, 5-HT_2A_R-sensitive mechanisms may preferentially influence seizure maintenance/termination, whereas other lisuride-sensitive mechanisms, potentially involving 5-HT_1A_Rs, may contribute to seizure initiation. However, this mechanistic interpretation remains speculative and requires confirmation using selective receptor antagonists.

Behavioral state may also contribute to the observed effects, as ASs are vigilance-dependent [36,37]. Although transient changes in locomotor activity were qualitatively observed following lisuride administration, these were not systematically assessed. Importantly, ASs were identified by the simultaneous occurrence of electrographic SWDs and behavioral arrest, confirmed by video recordings. Nevertheless, the lack of systematic assessment of locomotor activity and vigilance states prevents us from fully excluding an indirect contribution of behavioral state to the observed drug effects.

In conclusion, lisuride’s biphasic effects on ASs may reflect a complex interplay among distinct neurotransmitter systems, receptor-mediated mechanisms, and thalamocortical network states. The early anti-absence effect appears to be largely 5-HT_2A_R-independent and may involve dopaminergic mechanisms that reduce both SWD occurrence and duration. In contrast, the delayed pro-absence response is characterized primarily by SWD prolongation and involves a 5-HT_2A_R-sensitive component. These findings are consistent with partially dissociable mechanisms governing SWD initiation and maintenance/termination and highlight how the effects of multi-target drugs such as lisuride may depend on interactions among receptor systems and thalamocortical network states.

## 4. Materials and Methods

### 4.1. Animals

Adult male GAERS (>P90) were bred from the colony at Cardiff University. Animals were provided with standard diet and water ad libitum and maintained on a 12:12-h light–dark cycle (lights on at 07:00). All procedures were performed in accordance with the UK Animals (Scientific Procedures) Act (1986), ARRIVE guidelines, Basel declaration including the 3R concept, the European Directive 2010/63/EU, and local ethical requirements. All efforts were made to minimize animal suffering and the number of animals used [38].

### 4.2. EEG Recordings

GAERS were chronically implanted with cortical EEG electrodes under isoflurane anesthesia. Rats received meloxicam (0.5 mg/kg) postoperatively and were allowed to recover for at least 7 days before EEG recordings were performed in freely moving animals. Animals were habituated to the recording environment before pharmacological experiments [5,8,9,39].

### 4.3. Pharmacological Treatments

Following a 1-h habituation period, a stable 1-h baseline EEG and video recordings were obtained before drug administration, followed by continuous EEG and video monitoring for 120 min after treatment. Lisuride was administered intraperitoneally (i.p.) at 0.1 or 0.5 mg/kg. These doses were selected with reference to the range previously investigated in GAERS [21].

To investigate the involvement of 5-HT_2A_Rs, the effect of lisuride (0.5 mg/kg, i.p.) was examined following a 10-min pretreatment with the selective 5-HT_2A_R antagonist MDL11,939 (0.5 mg/kg, i.p.). This protocol was based on our previous study in GAERS, which demonstrated that MDL11,939 administered at the same dose (0.5 mg/kg, i.p.) and with the same pretreatment interval effectively antagonized the anti-absence effects of the 5-HT_2A_R agonist TCB-2 [8]. Appropriate vehicle-treated animals were included according to the same experimental design. In all experiments, animals were randomly assigned to the different treatment groups.

### 4.4. Analysis of Absence Seizures

SWDs were initially detected semi-automatically using the SeizureDetect script in Spike2 software (Cambridge Electronic Design, Cambridge, UK), applying a threshold of ±5 standard deviations relative to the interictal EEG baseline. Candidate events were required to contain at least five spikes, last at least 1 s, and exhibit a characteristic rhythmic frequency of 5–12 Hz. All detected events were subsequently verified by visual inspection.

Spontaneous ASs were identified according to established criteria for GAERS [8], based on the occurrence of electrographic SWDs and concomitant behavioral arrest, as confirmed by simultaneous video recordings [7].

Seizure activity was quantified using three complementary parameters: total time spent in seizure, average seizure length, and seizure number. Total time spent in seizure was defined as the cumulative duration (in seconds) of all AS episodes occurring within each 20-min recording interval, calculated by summing the durations of individual seizures. Average seizure duration was calculated by dividing the total time spent in seizure by the number of AS episodes recorded within the same interval. Seizure number was defined as the total number of AS episodes occurring within each interval. Data were analyzed in consecutive 20-min intervals during the control period and throughout the 120-min post-treatment period. For each animal, all three seizure parameters were normalized to the mean value obtained during the pre-treatment control period (−40 to 0 min), comprising two consecutive 20-min intervals. This individual baseline was defined as 100%, independently of treatment group, so that values above or below 100% indicate an increase or decrease relative to each animal’s own baseline rather than to the vehicle-treated group. This temporal analysis allowed the identification of time-dependent effects that might be obscured by averaging seizure activity across the entire post-treatment period.

### 4.5. Statistical Analysis

For GAERS, the effects of systemic administration of the compounds were analyzed using GraphPad Prism version 7 (GraphPad Software, San Diego, CA, USA). Data were analyzed by two-way ANOVA, with drug treatment and time as factors. Dunnett’s multiple-comparisons test was used, where appropriate, to compare each drug treatment with the vehicle for the main treatment effect over the full treatment period and for simple treatment effects at individual 20-min time bins. Time spent in seizures, mean seizure duration, and seizure number were analyzed separately. Data are presented as mean ± SEM. Statistical significance was set at *p* < 0.05. Full statistical results are provided in Supplementary Table S1.

## Data Availability

The original contributions presented in this study are included in the article/Supplementary Materials. Further inquiries can be directed to the corresponding authors.

## Supplementary Materials

The following supporting information can be downloaded at: https://www.mdpi.com/article/10.3390/ijms27219815/s1.

## Abbreviations

The following abbreviations are used in this manuscript:

5-HT	5-Hydroxytryptamine (serotonin)
5-HT1AR	Serotonin 1A receptor
5-HT2AR	Serotonin 2A receptor
5-HT2CR	Serotonin 2C receptor
ANOVA	Analysis of variance
ARRIVE	Animal Research: Reporting of In Vivo Experiments
AS	Absence seizure
EEG	Electroencephalography
GABA	γ-Aminobutyric acid
GABAA	Type A γ-aminobutyric acid receptor
GAERS	Genetic Absence Epilepsy Rats from Strasbourg
i.p.	Intraperitoneal
SEM	Standard error of the mean
SWD	Spike-and-wave discharge
TC	Thalamocortical
VB	Ventrobasal thalamus
