# Supplementary material for: Biphasic Modulation of Absence Seizures by Lisuride in GAERS: Dissecting the Role of 5-HT2A Receptors
Source: Int J Mol Sci. 2026 Nov 1;27(21):9815. doi: 10.3390/ijms27219815 (PMC13762907; doi:10.3390/ijms27219815)
Supplement: Supplementary file 1 [file ijms-27-09815-s001.zip › ijms-4625867-supplementary.pdf]

Biphasic Modulation of Absence Seizures by Lisuride in GAERS: Dissecting the Role of 5-HT2A Receptors

Marcello Venzi [1], Vincenzo Crunelli [1,2,*] and Giuseppe Di Giovanni [1,3,4,*]

[1] Neuroscience Division, School of Bioscience, Cardiff University, Museum Avenue, Cardiff CF10 3AX, UK; marcello.venzi@gmail.com
[2] Institute of Pharmacology and Neurosciences, Faculty of Medicine, University of Lisbon, 1649-028 Lisbon, Portugal
[3] College of Medicine, Korea University, Seoul 02841, Republic of Korea
[4] Department of Medical and Surgical Sciences, University of Magna Graecia, 88100 Catanzaro, Italy

*Correspondence: crunelli@cardiff.ac.uk (V.C.); giuseppe.digiovanni@unicz.it (G.D.G.)

Running title: Lisuride in Absence Seizures

Keywords
Absence epilepsy; GAERS; Thalamocortical network; Ventrobasal thalamus; Spike–wave discharges; In vivo electrophysiology

Supplementary Table S1. Summary statistics

A. Dose-response lisuride (systemic administration)

Parameter	Statistical analysis	Time (min)	0.1 mg/kg		0.5 mg/kg	
			% change	p-value	% change	p-value
**Time spent in seizures (cumulative time)**	Two-way ANOVA (Drug)	–	F(2,144) = 5.20; p = 0.0066			
	Two-way ANOVA (Time)	–	F(5,144) = 1.60; p = 0.163			
	Two-way ANOVA (Drug × Time)	–	F(10,144) = 8.57; p < 0.0001			
	Main treatment effect (full period)	SME	**31.9**	*	—	—
		20	78.7	ns	−99.2	*
		40	19.6	ns	−82.2	*
		60	19.4	ns	−13.4	ns
		80	35.2	ns	12.3	ns
		100	36.1	ns	55.2	ns
		120	−3.0	ns	118.4	*
**Mean seizure duration (length)**	Two-way ANOVA (Drug)	–	F(2,144) = 4.37; p = 0.014			
	Two-way ANOVA (Time)	–	F(5,144) = 5.83; p < 0.0001			
	Two-way ANOVA (Drug × Time)	–	F(10,144) = 5.86; p < 0.0001			
	Main treatment effect (full period)	SME	**26.9**	*	—	—
		20	1.7	ns	−85.1	**
		40	−6.1	ns	−44.2	ns
		60	12.8	ns	−15.4	ns
		80	90.7	**	19.1	ns
		100	54.3	ns	55.2	ns
		120	17.1	ns	101.9	**
**Seizure number**	Two-way ANOVA (Drug)	–	F(2,144) = 0.76; p = 0.470			
	Two-way ANOVA (Time)	–	F(5,144) = 0.57; p = 0.726			
	Two-way ANOVA (Drug × Time)	–	F(10,144) = 2.80; p = 0.0034			
	Main treatment effect (full period)	SME	**6.44**	ns	—	—
		20	66.7	ns	−95.5	ns
		40	27.5	ns	−47.0	ns
		60	−7.5	ns	126.3	ns
		80	−26.2	ns	22.0	ns
		100	−13.9	ns	20.9	ns
		120	−9.0	ns	12.9	ns

B. Lisuride ± MDL11,939 (systemic administration)

Parameter	Statistical analysis	Time (min)	0.5 mg/kg lisuride		0.5 mg/kg lisuride + 0.5 mg/kg MDL11,939		0.5 mg/kg MDL11,939	
			% change	p-value	% change	p-value	% change	p-value
**Time spent in seizures (cumulative time)**	Two-way ANOVA (Drug)	–	F(3,192) = 10.53; p < 0.0001					
	Two-way ANOVA (Time)	–	F(5,192) = 2.87; p = 0.016					
	Two-way ANOVA (Drug × Time)	–	F(15,192) = 7.99; p < 0.0001					
	Main treatment effect (full period)	SME	**−8.4**	ns	**−34.4**	*	**22.8**	ns
		20	−99.2	**	−82.2	*	40.3	ns
		40	−83.6	***	−54.4	*	13.9	ns
		60	−4.5	ns	82.0	ns	48.8	ns
		80	13.4	ns	17.7	ns	32.6	ns
		100	52.9	ns	−59.3	ns	−54.4	ns
		120	127.0	***	−85.4	*	−4.6	ns
**Mean seizure duration (length)**	Two-way ANOVA (Drug)	–	F(3,192) = 28.60; p < 0.0001					
	Two-way ANOVA (Time)	–	F(5,192) = 3.33; p = 0.0066					
	Two-way ANOVA (Drug × Time)	–	F(15,192) = 4.19; p < 0.0001					
	Main treatment effect (full period)	SME	**5.3**	ns	**−49.0**	***	**39.4**	**
		20	−85.0	*	−74.6	*	46.1	ns
		40	−45.7	ns	−56.2	ns	32.4	ns
		60	−26.7	ns	−47.4	ns	31.3	ns
		80	18.3	ns	−21.9	ns	44.6	ns
		100	52.9	ns	−20.4	ns	−56.2	ns
		120	95.0	**	−73.0	*	27.7	ns
**Seizure number**	Two-way ANOVA (Drug)	–	F(3,192) = 3.06; p = 0.029					
	Two-way ANOVA (Time)	–	F(5,192) = 8.95; p < 0.0001					
	Two-way ANOVA (Drug × Time)	–	F(15,192) = 4.50; p < 0.0001					
	Main treatment effect (full period)	SME	**−4.9**	ns	**34.2**	ns	**−5.2**	ns
		20	−95.5	ns	−60.4	ns	3.7	ns
		40	−48.7	ns	24.9	ns	−10.0	ns
		60	56.2	ns	257.2	****	7.7	ns
		80	29.8	ns	93.2	ns	−13.3	ns
		100	25.0	ns	−54.9	ns	24.9	ns
		120	21.2	ns	−56.0	ns	−32.0	ns

Statistical analysis of the effects of systemic lisuride and MDL11,939 administration on ASs parameters in GAERS. Time spent in seizures, mean seizure duration, and seizure number were analyzed separately using non-repeated-measures two-way ANOVA, with drug treatment and time as factors for dose-responses lisuride **(A)** and Lisuride and MDL11,939 **(B)**. Dunnett's multiple-comparisons test was used to assess the simple main effect of each drug treatment versus vehicle over the full treatment period and at individual 20-min time bins. Data are expressed as percentage change relative to vehicle; $n = 9$ animals per group. Significance levels were defined as $*p < 0.05$; $**p < 0.01$; $***p < 0.001$ and $****p < 0.0001$; ns, not significant.
